# Tumor Microenvironment Evaluation for Gastrointestinal Cancer in the Era of Immunotherapy and Machine Learning

**DOI:** 10.3389/fimmu.2022.819807

**Published:** 2022-05-04

**Authors:** Zilan Ye, Dongqiang Zeng, Rui Zhou, Min Shi, Wangjun Liao

**Affiliations:** Department of Oncology, Nanfang Hospital, Southern Medical University, Guangzhou, China

**Keywords:** tumor microenvironment, gastrointestinal cancer, immunotherapy, chemotherapy, machine learning

## Abstract

A dynamic and mutualistic interplay between tumor cells and the surrounding tumor microenvironment (TME) triggered the initiation, progression, metastasis, and therapy response of solid tumors. Recent clinical breakthroughs in immunotherapy for gastrointestinal cancer conferred considerable attention to the estimation of TME, and the maturity of next-generation sequencing (NGS)-based technology contributed to the availability of increasing datasets and computational toolbox for deciphering TME compartments. In the current review, we demonstrated the components of TME, multiple methodologies involved in TME detection, and prognostic and predictive TME signatures derived from corresponding methods for gastrointestinal cancer. The TME evaluation comprises traditional, radiomics, and NGS-based high-throughput methodologies, and the computational algorithms are comprehensively discussed. Moreover, we systemically elucidated the existing TME-relevant signatures in the prognostic, chemotherapeutic, and immunotherapeutic settings. Collectively, we highlighted the clinical and technological advances in TME estimation for clinical translation and anticipated that TME-associated biomarkers may be promising in optimizing the future precision treatment for gastrointestinal cancer.

## Introduction

**Figure 1:**
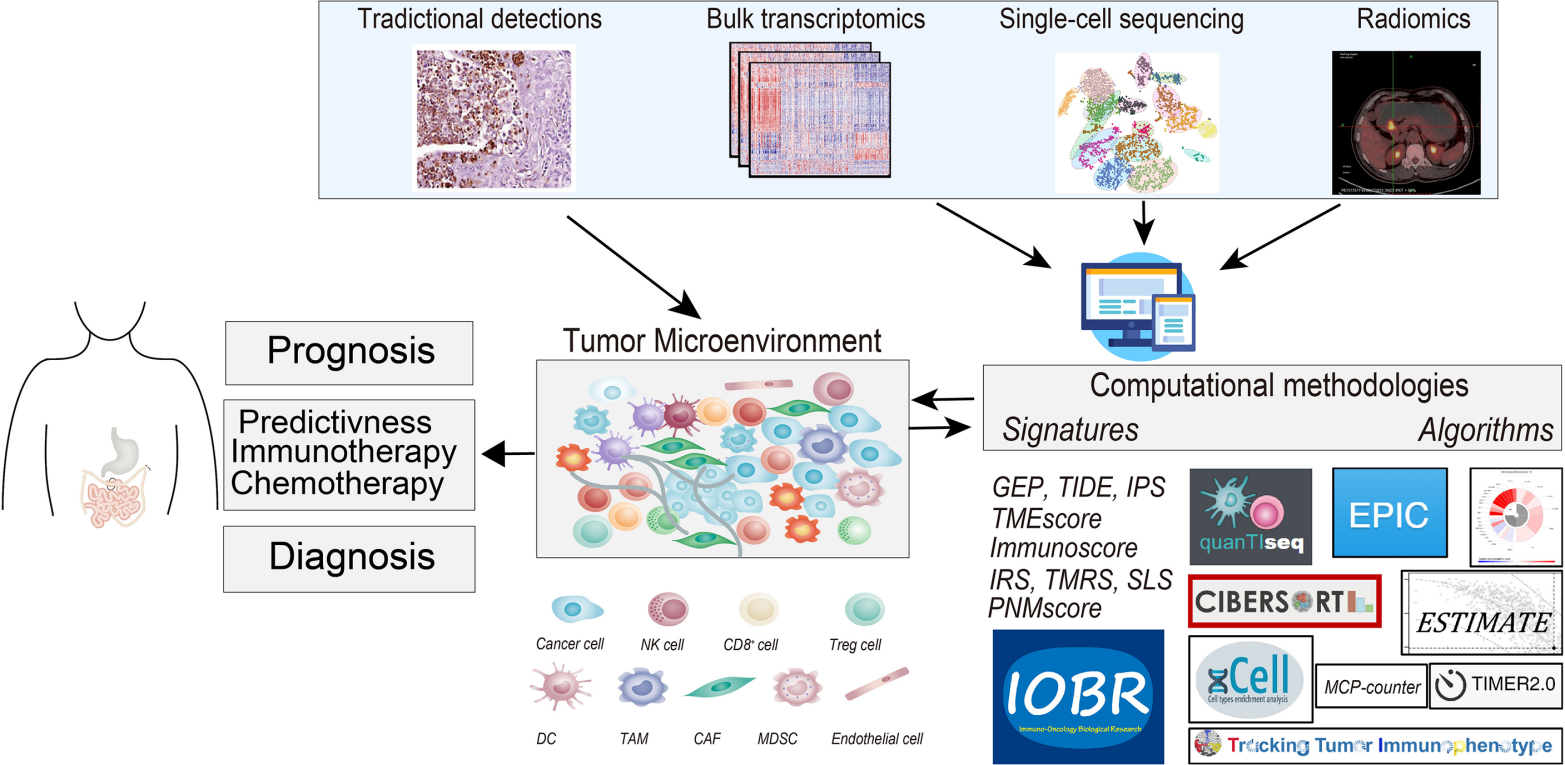
The graphical abstract depicts the tumor microenvironment (TME) in gastrointestinal cancer, outlines the methodologies for deciphering TME compartments, and elucidates the existing TME-relevant biomarkers in the prognostic, chemotherapeutic, and immunotherapeutic settings. Methods for TME assessment comprised the immunohistochemistry (IHC), the computational toolbox for NGS-based analyses, and radiomics detections. NK cell, natural killer cell; Treg cell, regulatory T cells; DC, dendritic cell; TAM, tumor-associated macrophages; CAF, cancer-associated fibroblasts; MDSC, myeloid-derived suppressor cell; NGS, next-generation sequencing.

The tumor microenvironment (TME) is a key component of tumor tissue and a vital determinant of tumor evolution and therapeutic response. Apart from tumor cells, TME is primarily comprised of tumor-infiltrating lymphocytes (TILs) (1), tumor-associated neutrophils (TANs) (2), tumor-associated macrophages (TAMs) (3), cancer-associated fibroblasts (CAFs) (4), endothelial cells, extracellular matrix proteins, associated inflammatory pathways (5), a variety of growth factors, chemokines, proteolytic enzymes, and specific biochemical characteristics, such as hypoxia and low pH (6) (**Figure 1**). The presence of a robust antitumor milieu characterized by a high infiltration of CD8^+^ cytotoxic T cells, Th1 helper cells, and memory CD45RO^+^ cells; expression of cytokines; and macrophage polarization toward M1 (3) often indicates a positive prognosis and can even lead to tumor elimination. However, elevated TGFβ levels (7), Treg cells (8), and fibroblast (9) infiltration often indicate poor prognosis. Some of the abovementioned features have been combined into an Immunoscore, which was elucidated as a putative predictor for prognosis and immunotherapeutic response in malignant tumors (6).

In the past few decades, cancer research attached its focus to the tumor cell, and insufficient attention has been focused on the crucial role of TME in carcinogenesis and progression. With the revolutionary breakthroughs in cancer immunotherapy including immune checkpoint blockades (ICBs), the evergrowing investigation into the host immune response has shed new light on the TME (10–15). Subsequently, extensive evidence has supported its prognostic value as well as the predictive significance in therapeutic response, covering immunotherapy (10, 16), targeted therapy (17), and chemotherapy (18). Moreover, the development of emerging technologies also facilitates unveiling TME and paving the way for individual medicine (19). To note, a recent comprehensive study developed a brand new TME signature, i.e., TMEscore, based on the clinical and genome-related data. The research deciphered the pattern of infiltrating cells and corresponding pathways within TME to construct the TMEscore as a robust predictive biomarker for prognosis and efficacy of ICBs in gastric cancer (6, 10) and other cancer settings.

As discussed in prior literature, intratumor heterogeneity (ITH) (20) may be the main barrier challenging the precise interpretation of TME-relevant biomarkers for clinical translation (21). Despite the well-constructed molecular subtypes in gastric cancer (22–24), comprising Epstein–Barr virus (EBV)-infected tumors, microsatellite instability (MSI), genome stability and chromosomally instability, and consensus molecular subtype (CMS) (25) in colorectal cancer (CRC), ITH may induce distinct molecular signatures in a diverse population of cells inter- or intratumors, such as the distinct immune landscape and treatment sensitivity of CRC in different anatomical locations.

Nevertheless, signatures derived from TME are still promising in predicting prognosis and improving individual medicine in clinical oncological practice; and comprehensive research and systematic review of the research to deepen our understanding of TME are still urgently needed. Here, we elaborate on the current understanding of the TME and the histological, radiomics, and emerging computational methodologies to construct TME-related signatures and the corresponding prognostic and predictive significance. The comparison and correlations among distinct predictive models are comprehensively discussed.

## Tumor Microenvironment Assessment Systems in the Age of Immunotherapy

### Clinical Utility of Traditional Methods in Tumor Microenvironment Assessment

Currently, the tumor–node–metastasis (TNM) staging system and histologic classification are used for routine prognostication and treatment among patients with gastrointestinal cancer, but neither of them functions well in TME estimation and clinical-outcome prediction (18, 26). To tackle this, immunohistochemistry (IHC), a typically cost-effective and clinical pragmatic method to identify different subpopulations of tumor-infiltrating cells, had been widely utilized in the generation of signatures to meet the clinical need for diagnosing and predicting specific therapeutic efficacy (26–29). However, the insufficient biopsy specimens, the heavy reliance on a limited repertoire of phenotypic markers, and interobserver bias have contributed to the irreproducible measurement and limitations of IHC in practical implementation and clinical standardization.

The multi-spectral imaging analysis techniques as a natural evolution of the conventional IHC method are promising in the context of preclinical experiments with the merit of precise immuno-colocalization and identification of cell interactions. Multiplex IHC/immunofluorescence (mIHC/IF) was leveraged to demonstrate that overexpression of SLAMF8 in macrophages may predict better anti-programmed cell death protein 1 (anti‐PD-1) immunotherapy efficacy in gastrointestinal cancer. However, its implementation in dissecting TME heterogeneity for predicting the therapy response of gastrointestinal cancer is limited (30). The technological challenges, high costs, and low throughput could be the primary obstacles that delay the clinical transition of multi-spectral imaging analysis.

Despite the existing restrictions, IHC is still well-adopted in signature construction. The IHC-estimated Immunoscore is recognized by the European Society for Medical Oncology (ESMO) clinical practice guideline for early-stage CRC (31). The consensus Immunoscore is one of the IHC-based scoring systems to summarize the density of CD3^+^ and CD8^+^ T-cell effectors within the central tumor and its invasive margin (29) and promisingly predict recurrence-free survival (RFS) of patients with colon cancer (29). Moreover, an IHC-reliant stromal signature, by estimating leukocyte and stromal factors, is also constructed for its prognostic value in estimating progression-free survival (PFS) of patients with resected pancreatic ductal adenocarcinoma (28). Additionally, another research constructed a risk signature, based on estimating the TME landscape of tumor-infiltrating inflammatory and immune cells by using IHC, and validated its correlation with poor RFS and chemotherapy resistance in patients with extrahepatic cholangiocarcinoma (27). Collectively, IHC has witnessed a collection of TME-related predictive signatures springing up in the preclinical studies.

Moreover, some other conventional methods like flow cytometry are also used for complementation (28). Take for example the aforementioned stromal signature constructed *via* IHC; the leukocyte cell population *via* flow cytometry (fluorescence-activated cell sorting) has functioned as a supplementary validation of the IHC evaluation, and the intratumoral leukocytes determined by it revealed a strong positive correlation with IHC staining on tissue blocks (28). Additionally, classical flow cytometry could work independently to identify the TME compositions of tumors such as non-small cell lung cancer to facilitate immune checkpoint inhibitor therapy clinical trial (32).

### Radiomics Interfaced With Tumor Microenvironment Evaluation

In the past decade, the field of medical image analysis has grown exponentially, radiomics is well-recognized as a novel form of data reflecting not only macroscopic but also cellular and molecular properties of tissues. The advances in radiomics facilitate high-throughput extraction of quantitative features and subsequent conversion of images into mineable data, which enables the interface between radiomics and TME estimation and improved treatment decisions (33–35).

A recently reported radiomics signature for intratumor and peritumor CD8 cells was capable of discriminating inflamed tumors from immune-desert tumors, and a high baseline radiomics score indicated an improved response for immunotherapy and prolonged overall survival (35). Considering the merit of non-invasiveness, radiomics is also implemented to improve the TME prognostic or predictive signatures derived from IHC detection. Li et al. developed an imaging signature for an Immunoscore, which was originally constructed utilizing IHC detection, by radiomics analysis of pretreatment CT images (34). Another study developed a radiological deep-learning signature for the non-invasive estimation of tumor stroma and adjuvant chemotherapy outcomes in patients with gastric cancer (33). The invasiveness of estimating tumor-infiltrating lymphoid and myeloid cells based on IHC staining of surgical specimens could be surmounted by the implementation of radiomics.

Collectively, non-invasive images are more than a visual interpretation of tumor tissue but data containing a spectrum of cellular and molecular information for evaluation of TME components and construction of signature for clinical translation and treatment-decision optimization.

### Computational Tools for Decoding Tumor Microenvironment Contexture From Multi-Omics Profiling

Instead of traditional IHC and radiomics, the increasing application of high-throughput next-generation sequencing (NGS) techniques for genome, transcriptome, or epigenome profiling has become the main source of data for cancer immunogenomics (36). Recent advances in computational algorithms and tools were leveraged to dissect the TME interaction including immune cells and stromal cells (37) (**Figure 1**). Tools for decoding immune contexture are currently classified according to four proposed computational principles: 1) machine learning-based principles, 2) gene set enrichment analysis (GSEA)-based principles, 3) linear regression-based principles, and 4) non-linear programming-based principles (38).

Among machine learning-based tools, CIBERSORT proposed by Newman et al. is the most popular methodology to refer to 22 immune cells in TME by adopting support vector regression for deconvolution, which enables large-scale analysis of RNA mixtures for cellular biomarkers and therapeutic targets with promising accuracy (39). However, the intrinsic limitation of deconvolution methods primarily lies in the fact that their performance is intrinsically tied to how well the reference expression matrix represents the gene expression of the immune cells (19). Though it may weaken the representability, CIBERSORT is still a robust computational tool to enumerate cell subsets, generating TME-associated predictive signatures (6, 11, 12). Moreover, the recently released CIBERSORTx expands its applicability to enumerate cell subsets and corresponding gene expression profiles from single-cell RNA-sequencing (RNA-seq) data (40). MethylCIBERSORT is also developed to address the restriction of CIBERSORT in genome-wide DNA methylation data for accurately determining tumor purity and cellular compositions (41).

Current GSEA-based computational tools include Tumor Immunology miner (TIminer) (42), Xcell (43), Estimation of STromal and Immune cells in MAlignant Tumour tissues using Expression data (ESTIMATE) (44), and Immune Cell Abundance Identifier (ImmunCellAI) (42). Notably, xCell is a gene signature-based web tool to estimate 64 immune cells from RNA-seq data and other cell subsets in bulk tumor tissue (43). Additionally, ESTIMATE infers non-tumor contexture including stromal and immune signatures for determining tumor purity (44). All four tools are available for transcriptomic data analysis, but TIminer is also suitable for DNA data (42).

Linear regression-based tools comprise Tumor Immune Estimation Resource (TIMER) (36), Estimating the Proportion of Immune and Cancer cells (EPIC) (45), Deconvolution for mixed cancer transcriptomes using raw measured data (Demix) (46), TIseq (47), MMAD (48), and Microenvironment Cell Populationscounte Microenvironment Cell Populations counter (MCP-counter) (37). TIMER is the first method that provides 6 major analytic modules, allowing integrative analysis of tumor immunologic, clinical, and genomics data (49). EPIC detects the proportion of immune and cancer cells from the expression of genes and compares it with the gene expression profiles from specific cells to predict the cell subpopulation landscape (45).

The non-linear programming-based principle is applied in Population-Specific Expression Analysis (PSEA) (50) and Digital Sorting Algorithm (DSA) and R package (51), which could extract specific gene expression profiles of distinct cell types without prior knowledge of their exact cell frequencies.

Additionally, the R package Immunedeconv integrates multiple existing microenvironmental deconvolution methodologies (52). Apart from TME deconvolution with diverse algorithms, the functions of another tool, namely, IOBR, cover as follows: multi-omics interpretation, including signature score calculation and estimation of signature–phenotype interactions, signatures derived from scRNA-seq data and genomic landscapes in multiple cancers, and fast signature construction (53).

Taken together, non-linear programming-based principles do not require the exact infiltration populations of different cell types as prior knowledge, whereas the other three principles rely on prior knowledge of marker genes of different immune cell subsets. Machine learning-based principles can computationally estimate the absolute proportion of immune cell infiltration in tumor tissue, whereas GSEA-based principles can infer the relative proportion of infiltrating immune cells in tumor tissue (38). Compared with three other deconvolution methods [linear least-squares regression (LLSR), quadratic programming (QP), and perturbation model for gene expression deconvolution (PERT)] under all the same test conditions, CIBERSORT is superior to other methods. EPIC and quanTIseq are the linear regression-based methods providing an “absolute score” that represents a cell fraction (38). Other methods provide scores in arbitrary units that are only meaningful in relation to another sample of the same dataset (52). Therefore, EPIC and quanTIseq are recommended for general purpose deconvolution, whereas MCP-counter as a relative scoring method is a good choice for clinical trial due to its highly specific signatures that excelled in the spillover benchmark (52). Collectively, the development and application of sophisticated computational tools assist in the selection of algorithms and assembly of analytical pipelines, shed light on tumor–host interaction, and facilitate the discovery of therapeutic targets and biomarkers for clinical translation (54).

### Tumor Microenvironment-Relevant Signatures Based on Big Data Research and Machine Learning

The irreversible wave of artificial intelligence and machine learning techniques gives impetus to current cancer research in TME using big data research. Based on the abovementioned methodologies, a collection of TME-relevant signatures was constructed to predict prognosis and individualize oncotherapy.

Considering prognosis, PNM score, Immunoscore proposed by Zeng et al. (11), TMEscore (10), and nomogram (55) are CIBERSORT-based prognostic signatures of gastric cancer by decoding the immune infiltration pattern with transcriptomic data. Additionally, TME-associated signatures, involving prognostic immune risk score (pIRS) (13), TME risk score (TMRS) (12), and CSS sets (56), are the predictive clinical outcome of patients with colon cancers. Moreover, prognostic models, such as T cell-inflamed gene expression profile (GEP) (5, 16), TIDE (57), and TMEscore (10), are reported also to be promising in pan-cancer settings (**Figure 1**).

The predictive value of most TME-related signatures is limited not only to prognosis but also to response to specific oncotherapy. The aforementioned GEP (5, 16), TIDE (57), TMEscore (10), and PNM score (58) are also robust biomarkers for response to immunotherapy. The Immunoscore proposed by Zeng et al. (11), nomogram (59), TMRS (12), CSS sets (56), and Tex (60) are predictive of sensitivity to adjuvant chemotherapy in specific gastrointestinal tumor settings. Despite evidence of the correlation between targeted therapy and immune modulation (61), fewer TME-relevant predictive signatures appear in this setting. The details on the predictive value of these models and their comparisons are discussed in the following sections.

Taken together, a combination of computational tools and transcriptomic data is a promising approach to overcome the technical limitations of IHC and identify diverse immune populations in a large patient cohort. In the future, putting forward the application of the efficient and emerging methodologies in current machine learning might facilitate an in-depth understanding of TME interaction and the immunotherapy-resistance mechanism to pave the way for individualized treatment of cancer.

### Promising Tools to Conquer Intratumor Tumor Microenvironment Heterogeneity

The ITH is one of the well-acknowledged challenges leading to treatment resistance (20) and impairing the accuracy of currently available predictors for diagnosis and therapeutic response (21). However, single-cell characterization based on sorting tumor-infiltrating cells and subsequent analysis, such as high-throughput flow cytometry (62) and single-cell sequencing (63), could be promising to tackle this challenge or effectively minimize its implication. In the past few years, the progress in single-cell isolation, nucleic acid amplification, and transcriptome profiling technologies (64) has led to novel single-cell sequencing (65), which will have a growing role in the future, in particular for the phenotyping of immune cells (66). However, despite the favorable merits, the technological challenges, sequencing costs, and practical restrictions are limiting the extensive utility.

### Comparison and Contrast of Tumor Microenvironment-Detection Methodologies in Clinical Practice

IHC as a typically cost-effective and clinical pragmatic method to identify different subpopulations of infiltrating cells within the TME had been widely utilized in the generation of signatures for clinical practice. However, the insufficient biopsy specimens, the heavy reliance on a limited repertoire of phenotypic markers, and interobserver bias remain the major obstacles to its practical implementation and clinical standardization. Compared with IHC, large-scale analysis of RNA mixtures for cellular biomarkers is featured with high throughput and promising accuracy. Adequate data-mature deconvolution methodologies may contribute to its clinical utility and consequently may accelerate data accumulation. However, signatures generated by bulk sequencing confront the well-acknowledged challenge of intratumor TME heterogeneity, which could be conquered by single-cell sequencing and multi-spectral imaging analysis such as mIHC/IF. Despite the high throughput and resolution of single-cell sequencing, the technological challenges, sequencing costs, inadequate data, and practical restrictions are limiting the clinical implementation. Multi-spectral imaging analysis as a natural evolution of the conventional IHC method is promising in the context of preclinical experiments with the merit of precise immuno-colocalization and identification of cell interactions but accompanied by the drawbacks of high costs and low throughput. Radiomics is implemented to improve the TME prognostic or predictive signatures derived from IHC detection, considering the merit of non-invasiveness and reflecting macroscopic properties of the tissue. However, insufficient computational resources and ITH may be the primary roadblock to its extensive utility.

## Prognostic Value of Models Derived From Tumor Microenvironment Assessment

### Prognosis Prediction Using Models Constructed *via* Traditional Immunohistochemistry Methods

Gastrointestinal cancer, especially gastric cancer, has large variations in the clinical prognosis even among patients with the same TNM stage (67). The heterogeneous property of gastric cancer requires comprehensively taking account of the outcome-relevant TME components and developing prognosis predictive models with better accuracy and efficiency to complement the TNM staging system.

To meet the need, several models *via* traditional IHC technologies were constructed (26, 29). Jiang et al. developed a novel model, named GC-SVM classifier. Taking account of either immune cell density or the tumor region, and with SVM algorithms to improve the accuracy, it comprehensively integrated information of eight TILs and myeloid cell IHC features (CD3IM, CD3CT, CD8IM, CD45ROCT, CD57IM, CD66bIM, CD68CT, and CD34), along with clinicopathologic features including patient sex, carcinoembryonic antigen (CEA) and lymph node metastasis (26). The study validated the GC-SVM classifier as an independent prognostic factor of overall survival (OS) and disease-free survival (DFS) in gastric cancer and a promising complement to the TNM staging system (26).

Additionally, Immunoscore, another prognostic model, is primarily proposed by Galon et al., integrating type, density, and location information of immune cells (68). Immunoscore immunohistochemically quantifies the *in situ* immune infiltration based on the enumeration of two lymphocyte populations (CD3/CD45RO, CD3/CD8, or CD8/CD45RO) in CRC (68) and provides a score ranging from Immunoscore 0 (I0) to Immunoscore 4 (I4) according to calculated immune-infiltration density (69). The five Immunoscore groups were associated with dramatic differences in DFS and OS and relapse in patients with early TNM stage CRC (70). Additionally, combined evidence supported its implementation as a new component in the classification of cancer, designated TNM-Immune (69), which predicts survival better than the TNM system alone (29).

Subsequently, Wang et al. first extended the feasibility of the aforementioned Immunoscore system to predicting RFS and OS in CRC liver metastases after liver metastasectomy (71). Additionally, Immunoscore was significantly and negatively associated with the clinical risk score (CRS) in which patients with more distant metastases presented a significantly lower density of lymphocytes (CD3, GZMB, CD8, T-Bet, CD57, and CD45RO) in tumors (72), despite that preoperative chemotherapy may impair the prognostic performance of the Immunoscore (71).

In a nutshell, despite the technological restrictions of IHC, the existing IHC-based TME estimation is still promising in predicting the clinical outcome of patients with gastrointestinal cancer. IHC-based estimation of immune infiltration may work well in supplementing the TNM staging to improve the classification system and to enhance the accuracy and efficiency of prognosis prediction, thereafter providing the impetus for individual medicine in cancer. Furthermore, the feasibility of these scoring models among other malignant tumors is under research and worth further clarification.

### Prognostic Models Developed Through Emerging Technologies

However, the technological restriction of IHC combined with the advances in high-throughput sequencing technologies and machine learning contributes to the advent of a spectrum of gene signature-based prognostic models in the setting of gastric cancer, CRC, and pan-cancer.

In terms of gastric cancer, Zeng et al. proposed that Immunoscore based on the fractions of 11 immune cells is a promising signature for estimating OS in patients with gastric cancer, using a least absolute shrinkage and selection operator (LASSO) Cox regression model (11). Notably, the follow-up study in gastric cancer, with the help of principal component analysis algorithms, has developed another robust prognostic and predictive biomarker termed TMEscore (10) and revealed its correlation with the intrinsic genome, metabolic characteristics, and molecular subtypes of gastric cancer (6, 22). The high TMEscore, which suggests prolonged overall survival, was markedly associated with immune activation and response to virus and IFN-γ, whereas the activation of transforming growth factor β, epithelial–mesenchymal transition, and angiogenesis pathways was enriched in the low TMEscore subgroup, which indicates T-cell suppression and may contribute to the poor prognosis in gastric cancer (10). Intriguingly, the subsequent analysis also indicated its potential application in pan-tumor prognostic prediction (6). Additionally, Lin et al. established an integrated PNM score system, based on RNA expression data, to predict the clinical survival of patients with gastric cancer, which comprises signatures of tumor protein-coding genes (P), tumor non-coding genes (N), and immune/stromal cells in the TME (M), a prognostic nomogram taking account of tumor size, site of origin, and mitotic index, accurately predicting RFS in the setting of post-resection localized primary gastrointestinal stromal tumors (GIST) (59). However, clinical translations of biomarkers that correlated with the efficacy of immunotherapy in gastrointestinal cancer are still lacking. Recently, a study applied a 395-plex immune-oncology (IO)-related gene profiling platform and machine learning strategy to determine a novel RNA signature that may reflect the “immune-responsive feature” of both cancer cells and the immune microenvironment, thereafter facilitating the patient selection for ICB treatment (73).

Regarding stage II CRC, combinatory cancer hallmark-based gene signature sets (CSS sets) were developed to identify patients with better 5-year RFS rates (56). Moreover, the TMRS (12) and pIRS (13) models are the latest proposed promising and novel signatures for prognosis prediction in terms of stage I–III colon cancer to complement TNM staging system and overcome the IHC-technical restriction by adopting transcriptomic data.

## Significance of Tumor Microenvironment-Relevant Models in Predicting Response to Adjuvant Chemotherapy

### Predictive Models Constructed *via* Traditional Immunohistochemistry Methods

Adjuvant chemotherapy has been recommended as a standard component of therapies for patients with stage II and III gastric cancer and improves their outcomes (74). However, only a subset of patients could benefit from adjuvant chemotherapy, and the criterion for the selection of candidates is still controversial (74). The aforementioned IHC-based prognostic models could also be a promising biomarker for adjuvant chemotherapy in gastrointestinal cancer.

A study of the GC-SVM classifier that identified both tumor-infiltrating immune and stromal cells revealed that adjuvant chemotherapy significantly increased OS and DFS in the high-gastric cancer-SVM group (26). In either stage II or III gastric cancer, the benefit from adjuvant chemotherapy was superior among patients with high GC-SVM (26). Before establishing the gastric cancer-SVM classifier, the team had proposed an ImmunoScore signature (IS_GC_) (18) by using IHC and another individualized predictive nomogram (55) to identify stage II–III gastric cancer patients who may derive clinical benefits from adjuvant chemotherapy. Moreover, their subsequent study developed a non-invasive imaging signature for IS_GC_ (RIS) by radiomics analysis of pretreatment CT images and corroborated the consistency between RIS and IS_GC_ (34). Recently, the N0417 trial also demonstrated that the microenvironmental features of TIL density and tumor budding are significant variables for DFS of patients treated in a phase III adjuvant trial of FOLFOX-based therapy and may determine colon tumor metastatic potential (75), which collectively suggests that the pivotal and complex tumor-TME cell crosstalk may also impact adjuvant chemotherapy regimens.

### Models Developed by Using Emerging Technologies and Its Role in Predicting Adjuvant Chemotherapy Benefits

Given the significant role that TME potentially plays in adjuvant chemotherapy, a collection of TME-related computational models also stepped into the field of response prediction, including part of the aforementioned prognostic signatures.

In the setting of gastric cancer, Lin et al. proposed the prognostic PNM score model and demonstrated that its combination with tumor mutational burden (TMB) could accurately predict sensitivity to tumor-suppressive adjuvant chemotherapy (58). Intriguingly, to some extent, adjuvant chemotherapy may conversely impair the capacity of some prognostic biomarkers like Immunoscore (11). However, in the setting of GIST, another prognostic nomogram established by Gold et al. is also suggested to be constructive for patient care, interpretation of clinical trial results, and selection of patients for adjuvant imatinib treatment (59).

In terms of stage II CRC after fluorouracil-based adjuvant chemotherapy, the CSS set may facilitate determining the treatment response. A substantial gain in survival benefits from adjuvant chemotherapy was observed in the low-risk patient group, with a reduction in recurrence by 30%–40% in 5 years. Moreover, with the significant prognostic value expected, the TMRS panel of CRC, based on TME genes, was reported to precisely predict the efficacy of adjuvant chemotherapy and tailor therapeutic strategies in a stage I–III setting, which partially provides an insight into the multidimensional information regarding the heterogeneity of TME.

## Tumor Microenvironment-Related Signatures That Facilitate Tailoring Immunotherapy

The occurrence of the immunotherapeutic era further encourages the researchers to dissect the TME heterogeneity and work in promoting its clinical translation to better tailor immunotherapy. The lack of robust biomarkers that predict beneficial and durable responses and avoid unnecessary and unfavorable drug adverse events is the major challenge encountered in current ICB therapy. Though programmed death ligand-1 (PD-L1) expression level, TMB, neo-antigen burden, copy-number alterations (CNAs) (76), mismatch repair deficiency, antigen presentation defects, intestinal microbiota, and molecular subtypes could influence ICB efficacy to a certain extent, none of them could independently and perfectly determine ICB response and resistance.

Nevertheless, benefiting from the digital analyzer, recent studies have indicated a collection of TME-related models for assessing immunotherapeutic benefits in pan-cancer. The T cell-inflamed GEP score (5) is either an individual cross-tumor predictor or combined with other predefined biomarkers like TMB (16), for immunotherapy, especially in PD-1 blockade- or PD-L1-based combination therapy regimens. Additionally, another pan-cancer scoring scheme, namely, immunophenoscore, was built using machine learning to identify determinants of tumor immunogenicity for response to anti-cytotoxic T lymphocyte antigen-4 (CTLA-4) and anti-PD-1 therapy (77). TIDE, a computational method developed by Peng Jiang et al. for pretreatment gene expression data, models two primary tumor immune-evasion mechanisms: the induction of T-cell dysfunction and the T-cell exclusion, which may contribute to its more precise prediction of patient response in the setting of first-line anti-PD-1 or anti-CTLA-4 treatment than PD-L1 level and TMB (57).

However, the biomarkers for immunotherapy in gastric cancer are relatively rare, but reports in recent years still yield an exciting breakthrough. In the PNM score system, Lin et al. evidenced that combination of the immune/stromal cells in TME signature, the protein-coding genes signature, and TMB would be a promising candidate to predict gastric cancer patients’ response to anti-PD-1/PD-L1 treatment (58).

Notably, the latest TMEscore proposed by Zeng et al. consistently supported the essential role that TME plays in ICB treatment of gastric cancer (6, 10). The TMEscore is elevated in MSI and EBV molecular subtypes (22–24), which have been suggested to be more sensitive to PD-1 immune-checkpoint blockade but could not completely explain the therapeutic response (6, 78) in that EBV^+^ tumors with low mutation burden could also exhibit immune infiltration (24, 78). However, TMEscore significantly elevated the predictive value for ICB therapy, relative to EBV or MSI status alone, which is potentially attributed to its close correlation with both EBV and MSI status. Furthermore, the intrinsic metabolic and genomic characteristics, as well as molecular subtype distribution of gastric cancer in different TMEscore subsets, were also discussed to explore the underlying TME-metabolic and TME-genome network, which confer the immunotherapeutic resistance or response (6). In this regard, two ongoing prospective observational studies are exploring its correlation with immunotherapeutic efficacy in the setting of advanced gastric cancer (NCT04850716) and the perioperative treatment of locally resectable adenocarcinoma of the esophagogastric junction or gastric cancer (NCT04850729). Collectively, taking TME signatures or the biomarker combination strategy into consideration may optimize the current biomarker system of gastric cancer to further advance precision immunotherapy (**Table 1**).

**Table 1:** The implementation and comparison of biomarkers based on TME evaluation.

Methodology	Cancer	Biomarkers	Prognosis	Chemotherapy response	Immunotherapy response
IHC	GC	GC-SVM classifier	Yes	Yes	
	GC	Immunoscore/TNM-Immune	Yes		
	GC	IS_GC_		Yes	
	CRC	CRS	Yes		
bulk-seq	GC	Immunoscore	Yes	Yes	
	GC	TMEscore	Yes		Yes
	GC	PNM score system	Yes	Yes	Yes
	GC/CRC	IO-score	Yes		Yes
	CRC	CSS sets	Yes	Yes	
	CRC	TMRS	Yes	Yes	
	CRC	pIRS	Yes		
	Pan-cancer	T cell-inflamed GEP score			Yes
	Pan-cancer	Immunophenoscore			Yes
	Pan-cancer	TIDE			Yes
Radiomics	GC	RIS		Yes	

TME, tumor microenvironment; IHC, immunohistochemistry; GEP, gene expression profile.

## Conclusions

The TME has profound clinical and therapeutic implications in gastrointestinal cancer. Comprehensive and quantitative interrogation of TME requires the employment of traditional molecular and cellular methodologies, as well as emerging computational tools for analyzing multi-omics data. The Immunoscore detected using IHC is adopted in the ESMO clinical practice guideline for early-stage CRC (31). However, the IHC is relatively labor-intensive, time-consuming, technically challenging, and not suitable for clinical applications, as compared to NGS technology.

The maturity of NGS-based technology triggered an ever-increasing sophisticated toolbox for RNA-seq and the availability of enormous amounts of datasets, which are essential prerequisites to extract information for precision cancer therapy and explore novel frontiers. Tumors are complex ecosystems comprised of diverse cell populations. The enumeration of TIL phenotypic diversity and composition in solid tumors recently attracted considerable interest. Immune deconvolution techniques extracted the data from RNA-seq or microarray, providing a more comprehensive landscape of the tumor and the microenvironment. Advances in single-cell technology allow TILs to be profiled with increasingly high resolution and accuracy. An in-depth understanding of TIL biology, both at crucial clinical milestones and in relation to therapy, would aid in the discovery of predictive and prognostic signatures and novel treatment targets. Clinical translation of robust biomarkers is pivotal for precision treatment interventions and optimized therapy regimens.

However, intertumoral heterogeneity poses considerable challenges to address the potential batch effects and fluctuating cutoff of gene signature scores in individual patients, which could be the primary obstacles that delay the clinical transition of most existing signatures from bench to bedside. Both challenges could be overcome by ceaselessly updating computational algorithms in the upcoming years. We anticipate that existing and future computational tools will be instrumental for detecting TME in individual patients and will ultimately facilitate precision treatment for malignant cancers, which provides a rationale for optimal combination of TME-associated biomarkers and other well-recognized biomarkers, or latent synergy among biomarkers derived from different omics.

## Outstanding Questions

The advances in clinical translation of TME estimation, such as Immunoscore and TMEscore for predicting therapeutic efficacy, pose new challenges to unbiased spatiotemporal assessment of microenvironment, which demands considerable research for sophisticated computational algorithms in the upcoming future by experts in computational biology and artificial intelligence. Despite the advent and development of single-cell and spatial transcriptomics providing insights into dynamic evolution and intercell crosstalk within TME with high resolution, the lack of adequate single-cell data and feasible methodologies remains an obstacle for decoding explicit phenotypical characterization of immune cell populations, and a more comprehensive and spatiotemporal landscape of TME contexture, as well as its correlation with clinical outcomes. Substantial research has evidenced the dynamic shift of local immune contexture from a preexisting to a therapy-induced immune response that accompanies high heterogeneity and plasticity, which highlights TME remolding as a crucial and novel orientation for inducing antitumor immunity. Currently, the insufficiency of large-scale and dynamic multi-omics cohorts under a uniform detection criterion is a major stumbling block to unraveling the actual and perplexing correlation between spatiotemporally heterogeneous TME and phenotypes of malignancies. Collectively, the active collaboration between computational biologists and physician-scientists, as well as the integration of multi-omics biological data and emerging artificial intelligence algorithms, warrants rational clinical translation of investigations of univocal TME characterizations along the process of tumorigenesis, progression, and response or resistance for antitumor therapy.

## Author Contributions

Drafting of the manuscript: ZY and DZ. Critical revision of the manuscript for important intellectual content: RZ, MS, and WL. Obtaining funding: WL. Administrative, technical, or material support: DZ. Supervision: WL. All authors listed have made a substantial, direct, and intellectual contribution to the work and approved it for publication.

## Funding

This work was supported by the National Natural Science Foundation of China (Nos. 82073303 and 81772580), the Guangzhou Planned Project of Science and Technology (No. 201803010070), and the Guangdong-Macao Science and Technology Innovation Joint Fund Project and Hong Kong-Macao Science and Technology Achievement Transformation Project in Guangdong (No. 2020A0505090007) for data collection and interpretation.

## Conflict of Interest

The authors declare that the research was conducted in the absence of any commercial or financial relationships that could be construed as a potential conflict of interest.

## Publisher’s Note

All claims expressed in this article are solely those of the authors and do not necessarily represent those of their affiliated organizations, or those of the publisher, the editors and the reviewers. Any product that may be evaluated in this article, or claim that may be made by its manufacturer, is not guaranteed or endorsed by the publisher.
